# A Clinical Prediction Model for Atypical Tuberculosis Manifestations Among Older Adults

**DOI:** 10.3390/medicina61101888

**Published:** 2025-10-21

**Authors:** Jun-Jun Yeh, Jia-Hong Chen, Yi-Ling Kuo, Chieh-Hsuan Tsai, Yung-En Ko

**Affiliations:** 1 Department of Thoracic Medicine, Family Medicine, Geriatric Medicine, Medical Research, and Medical Education, Ditmanson Medical Foundation Chia-Yi Christian Hospital, Chiayi 600566, Taiwan; 07888@cych.org.tw; 2 Department of Radiologic Medicine, Chest Medicine, and Medical Research, Ditmanson Medical Foundation Chia-Yi Christian Hospital, Chiayi 600566, Taiwan; 05564@cych.org.tw; 3 Department of Nutrition Medicine, Chest Medicine, and Medical Research, Ditmanson Medical Foundation Chia-Yi Christian Hospital, Chiayi 600566, Taiwan; 02079@cych.org.tw; 4 Department of Family Medicine, Chest Medicine, and Medical Research, Ditmanson Medical Foundation Chia-Yi Christian Hospital, Chiayi 600566, Taiwan; aurora90083@gmail.com

**Keywords:** tuberculosis, older adults, atypical presentation, TRIPOD, calibration, decision curve analysis, predictive model

## Abstract

*Background and Objectives:* Active pulmonary tuberculosis (aPTB) in the Older Adults (≥75 years) is frequently under-recognized in non-pulmonology settings due to atypical symptoms and multiple comorbidities. This study aimed to develop and validate a TRIPOD-compliant clinical prediction model for early identification of atypical aPTB in this vulnerable population. *Materials and Methods:* We retrospectively analyzed 5651 patients aged ≥75 years with culture-confirmed aPTB and World Health Organization (WHO) symptom scores < 5. Patients were stratified into Group a (Ga, Patients with aPTB not initially suspected by non-pulmonologists (atypical presentation, WHO/CDC 7-point score < 5, n = 1155) and Group b (Gb, Patients without aPTB within the first 24 h (non-TB comparators), n = 4496). Multivariate logistic regression identified independent predictors of delayed diagnosis. A weighted scoring system was derived from β-coefficients and validated in independent derivation (2000–2020) and temporal validation (2021–2023) cohorts. Model discrimination, calibration, and decision curve analysis (DCA) were assessed following TRIPOD standards. *Results*: Five independent predictors—age > 85 years (OR = 6.31, 95% CI = 5.31–8.72), hypoalbuminemia (OR = 4.10, 95% CI = 3.92–7.26), cardiovascular disease (OR = 3.32, 95% CI = 1.23–5.27), diabetes mellitus (OR = 2.03, 95% CI = 1.32–4.07), and predominant lower-lung field involvement (OR = 1.25,95% CI = 1.03–2.44)—were incorporated into the scoring model. Using a cutoff ≥ 7, the model achieved excellent performance across all cohorts (AUC 0.95–0.96; sensitivity 91–94%; specificity 97–99%). Calibration plots and DCA confirmed strong agreement and high net clinical benefit. Nearly 70% of atypical cases had symptom scores ≤ 1, lacking typical signs such as fever or cough. *Conclusions*: Oldest-old (>85 years) emerged as the strongest independent predictor of atypical TB, surpassing conventional frailty indicators such as sarcopenia or osteoporosis. The proposed score provides a simple, accurate, and validated tool for early detection of aPTB in non-pulmonology settings. Its integration into electronic medical records may reduce diagnostic delays and improve outcomes in this high-risk, late-elderly population.

## 1. Introduction

Tuberculosis (TB) remains a persistent public health challenge, especially in aging societies [1]. Despite global progress in TB control, older adults—particularly those aged ≥75 years—face heightened risk of underdiagnosis and poor outcomes due to atypical clinical presentations and frailty-related factors [2]. As the global population ages, the proportion of ‘Older Adults (≥75 years)’ individuals is steadily rising, necessitating tailored diagnostic strategies [2]. Late elderly patients with active pulmonary tuberculosis (aPTB) often present with few or nonspecific symptoms, making clinical recognition difficult. To address this, the World Health Organization (WHO) and Taiwan Centers for Disease Control (CDC) endorse a seven-point symptom scoring system, in which a score ≥ 5 indicates a typical presentation of aPTB [3,4,5,6]. However, many older adults exhibit scores < 5, classified as atypical or silent TB [3,4,5]. Such presentations can easily be mistaken for heart failure, aspiration pneumonia, or other chronic conditions, resulting in missed or delayed diagnosis and increased mortality [6,7].

The co-occurrence of chronic conditions such as diabetes mellitus, cardiovascular disease, and hypoalbuminemia further obscures PTB diagnosis in this population [8]. While several studies have addressed diagnostic issues in older adults with PTB, few have focused exclusively on patients aged ≥75 years, and none have prioritized age stratification as a core diagnostic variable in Asian cohorts [3].

This study aims to characterize the clinical, radiographic, and metabolic profiles of Older Adults (≥75 years) aPTB patients with atypical symptoms. It also investigates whether age >85 years—an extreme aging threshold—serves as a superior diagnostic predictor compared to traditional markers like osteoporosis or sarcopenia [3,8,9,10,11].

## 2. Materials and Methods

### 2.1. Derivation Cohort

#### 2.1.1. Patient Selection and Grouping

**Figure 1:**
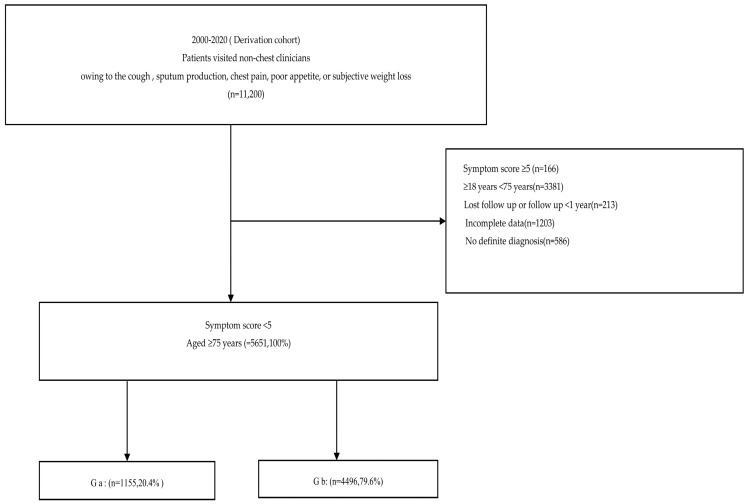
Older Adults (≥75 years) Diagnostic Stratification Process and Study Cohorts. Flowchart illustrating patient inclusion and stratification. Patients aged ≥ 75 years with culture-confirmed active pulmonary tuberculosis (aPTB) and WHO symptom scores < 5 were divided into Group a (Ga, Patients with aPTB not initially suspected by non-pulmonologists (atypical presentation, WHO/CDC 7-point score < 5, n = 1155) and Group b (Gb, Patients without aPTB within the first 24 h (non-TB comparators), n = 4496). Derivation (2000–2020) and validation cohorts (2021–2023) were used to evaluate the predictive model’s performance.

Patients in Chia-Yi, southern Taiwan, were classified based on symptom scores using the WHO/CDC-endorsed 7-point scale. Those with scores < 5 were defined as having atypical presentations [11,12]. Based on initial clinical impressions, patients were stratified into two groups: Group a, (Ga, Patients with aPTB not initially suspected by non-pulmonologists (atypical presentation, WHO/CDC 7-point score < 5, n = 1155) and Group b (Gb, Patients without aPTB within the first 24 h (non-TB comparators), n = 4496) (Figure 1, Table 1).

#### 2.1.2. Data Collection

The term ‘initial smear test’ refers to acid-fast bacilli (AFB) microscopy, which remains a standard first-line diagnostic tool for PTB in Taiwan. Although molecular rapid tests such as PCR (e.g., Xpert MTB/RIF) are available, they are primarily used for smear-positive cases to confirm diagnosis and detect rifampicin resistance [13]. For smear-negative cases, PCR testing is selectively applied based on clinical suspicion, radiographic findings, and physician discretion. This tiered approach reflects current practice patterns and resource allocation in Taiwan’s PTB diagnostic strategy. In this study, the microbiological confirmation of aPTB was based on mycobacterial culture results, which served as the reference standard. While AFB smear microscopy and nucleic acid amplification tests (NAATs) such as PCR were used as part of routine clinical evaluation, only cases with positive culture for *Mycobacterium tuberculosis* were included as microbiologically confirmed aPTB. Demographic variables included age, sex, and body mass index (BMI). Clinical information comprised presenting symptoms, sputum smear status, and initial diagnostic impressions. Medical histories captured comorbidities such as diabetes (DM), cardiovascular disease, hypoalbuminemia, chronic kidney disease, malignancy, liver disease, osteoporosis/sarcopenia, and a history of PTB.

#### 2.1.3. Imaging Assessment

Two radiologists and one pulmonologist, all blinded to clinical data and patient outcomes, independently evaluated each chest radiograph. Inter-observer agreement was assessed using Fleiss’ kappa statistic. Agreement was excellent, with a Fleiss κ = 0.91 (95% CI, 0.90–0.95); pairwise agreement between the two radiologists was κ = 0.95 (95% CI, 0.93–0.98). No intra-observer assessment was performed.

#### 2.1.4. Statistical Analysis

Continuous variables were expressed as mean ± standard deviation (SD) and compared using Student’s *t*-test. Categorical variables were analyzed using the Chi-square test or Fisher’s exact test, as appropriate. Odds ratios (OR), beta coefficients (β), and 95% confidence intervals (CI) were calculated. Analyses were conducted using SPSS v22.0. A weighted clinical scoring system was constructed based on the β coefficients derived from the final regression model. Following the method proposed by recent research, the smallest coefficient was assigned a score of 1, with other variables scaled proportionally (i.e., scores ranging from 2 to 5 based on magnitude) [14]. The discriminatory performance of the model was evaluated using receiver operating characteristic (ROC) curve analysis, and the area under the curve (AUC) was calculated to determine optimal cut-off points. Multivariate logistic regression was applied to identify independent predictors, and adjusted odds ratios (aOR) with 95% CI were reported. Discrimination was assessed using ROC curves and the AUC. Calibration was examined by the Hosmer–Lemeshow test and calibration plots. Internal validation was performed with 1000 bootstrap replications to account for model optimism. Clinical utility was evaluated using decision curve analysis (DCA). Collinearity among predictors was assessed with variance inflation factors (VIF), and model simplification was explored when high correlations were detected [5,8,15]. Post-test diagnostic accuracy was further evaluated using model sensitivity, specificity, pre-test disease prevalence, and likelihood ratios (LR^+^), allowing for the calculation of post-test probabilities to assess clinical utility.

### 2.2. Methods

#### 2.2.1. Validation Cohort

A temporal internal validation cohort, spanning from January 2021 to December 2023 (n = 998), underwent the same procedures as the derivation cohort, including regression modeling, score calculation, and evaluation of diagnostic performance.

#### 2.2.2. Subgroup Analyses

To evaluate the robustness and generalizability of the predictive scoring system, we conducted a temporal internal validation (2021–2023 cohort). Subgroup analyses included ages 75–85 years, ≥85 years, and patients without diabetes. The primary comparison was between patients with atypical manifestations of PTB and those without PTB. The following subgroup analyses were performed: (1) Cohort of Older Adults Aged 75–85 Years: This subgroup focused on patients aged between 75 and 85 years to determine whether the scoring system maintained discriminatory power in this specific elderly population, excluding the oldest-old (>85 years). This analysis addresses the diagnostic challenges often seen in late-elderly patients with atypical presentations. (2) Score System Excluding the oldest-old. To eliminate the potential confounding effect of extreme age on the scoring system, we conducted a parallel validation excluding the oldest-old. This subgroup helps evaluate the model’s performance in a younger geriatric population with potentially more classic disease manifestations. (3) Cohort Without DM: DM is a known modifier of PTB presentation. Therefore, we examined the score’s diagnostic performance in a non-diabetic population to assess whether its predictive value remains consistent in the absence of this common comorbidity (Table 2 and Table 3, Supplemental Tables S2–S4).

#### 2.2.3. Ethical Approval

This study is not a human trial of new drugs, new medical devices or new medical technologies. As the medical records were retrospectively reviewed, no identifying information was shown to identify the patient. Hence, obtaining informed consent from the patient was waived. All data were acquired from Chia-Yi Christian Hospital and branch hospitals in southern Taiwan. The study was approved by the Chia-Yi Christian Hospital Institutional Review Board (CYCH-IRB-2023078).

## 3. Results

A total of 11,200 subjects initially evaluated outside the chest department or by non-chest physicians were screened. To focus on atypical or silent presentations of PTB, individuals with WHO-defined symptom scores ≥ 5 were excluded. The final cohort comprised patients aged ≥ 75 years with a symptom score < 5. These patients were further stratified into two late-elderly subgroups based on final diagnosis: Ga and Gb. The derivation cohort contained 1155 events with five predictors, yielding an EPV of 231 (>20).

### 3.1. Baseline Characteristics

As shown in Table 1, patients in the Ga group were significantly older than those in the Gb group (mean age 90.87 vs. 81.92 years, *p* < 0.001), with 78.5% oldest-old. Ga patients also had higher rates of hypoalbuminemia (91.7%), diabetes (80.1%), cardiovascular disease (73.3%), and lower lung field lesions (81.7%) compared to Gb. Conversely, classic TB symptoms like fever and hemoptysis were less common in the Ga. Notably, 69.0% of Ga patients had a symptom score ≤ 1, highlighting the prevalence of atypical presentations.

### 3.2. Screening Potential Risk Factors

To identify potential predictors associated with delayed identification of aPTB in Older Adults (≥75 years) patients, we first considered the presence of concomitant aPTB as the dependent variable, while incorporating demographic, serological, and clinical features as independent variables. To address potential multicollinearity and improve model parsimony, least absolute shrinkage and selection operator (LASSO) regression was applied with ten-fold cross-validation to determine the optimal penalty coefficient (λ). 17 Univariate analyses were then performed to compare patients with Ga and Gb. Continuous variables were expressed as mean ± standard deviation and analyzed using the Student’s *t*-test, whereas categorical variables were compared using the χ^2^ or Fisher’s exact test. As summarized in Table 1, patients in Ga were significantly older than those in Gb (mean age 90.87 ± 6.03 vs. 81.92 ± 2.50 years, *p* < 0.001), with 78.5% oldest-old. The Ga also exhibited higher proportions of frailty-related comorbidities, including hypoalbuminemia (91.7% vs. 21.8%, *p* < 0.001), diabetes mellitus (80.1% vs. 22.7%, *p* < 0.001), cardiovascular disease (73.3% vs. 17.3%, *p* < 0.001), and osteoporosis/sarcopenia (73.1% vs. 16.7%, *p* < 0.001). In contrast, classical TB symptoms such as fever and hemoptysis were less frequent (22.9% vs. 45.0% and 38.6% vs. 52.0%, respectively), and 69.0% of Ga patients presented with a WHO symptom score ≤ 1, indicating profoundly atypical clinical manifestations. Lifestyle and hematological parameters further differentiated the groups. Smoking (42.9% vs. 14.2%, *p* = 0.021) and alcohol consumption (22.9% vs. 17.8%, *p* < 0.001) were more common in Ga. Laboratory markers revealed significant hypoalbuminemia, prolonged coagulation profiles, and elevated inflammatory indices among Ga patients, consistent with systemic frailty and metabolic disturbance. Radiological assessment demonstrated distinct imaging patterns. Lower-lung field predominance was markedly more frequent in Ga (81.7% vs. 30.6%, *p* < 0.001), whereas typical cavitation was substantially reduced (8.8% vs. 34.3%, *p* < 0.001). Non-nodular patches, interstitial or linear infiltrations, pleural effusion, and extrapulmonary lesions were also significantly enriched in Ga (all *p* < 0.001). Based on LASSO feature selection and univariate screening (*p* < 0.05), the following candidate variables were retained for multivariate logistic regression: oldest-old, hypoalbuminemia, diabetes mellitus, cardiovascular disease, osteoporosis/sarcopenia, and lower-lung field involvement. (Supplemental Figure S1). These parameters were subsequently entered into the multivariate model to identify independent predictors of Ga diagnosis (Table 2).

### 3.3. Multivariate Logistic Regression

Variables that reached statistical significance in univariate analysis (*p* < 0.05) were subsequently entered into a multivariate logistic regression model to identify independent predictors of delayed identification of aPTB among Older adult (≥75 years) patients. Stepwise selection was applied to optimize model fit and eliminate redundant variables. The variance inflation factors (VIFs) for all retained predictors were <5, indicating the absence of problematic multicollinearity.

As summarized in Table 2, five variables emerged as statistically significant independent predictors of delayed aPTB diagnosis in the Ga. The strongest predictor was oldest- old (β = 2.063, OR = 6.31, 95% CI = 5.31–8.72, *p* < 0.001), followed by hypoalbuminemia (<3.5 g/dL) (β = 1.562, OR = 4.10, 95% CI = 3.92–7.26, *p* < 0.001), cardiovascular disease (β = 1.210, OR = 3.32, 95% CI = 1.23–5.27, *p* = 0.001), diabetes mellitus (β = 0.825, OR = 2.03, 95% CI = 1.32–4.07, *p* = 0.020), and predominant lower-lung field involvement (β = 0.508, OR = 1.25, 95% CI = 1.03–2.44, *p* = 0.014).

Each β-coefficient was then converted to an integer weighting score based on its relative magnitude, following the proportional-scaling method proposed in prior diagnostic model studies. The smallest coefficient (lower-lung field involvement) was assigned a score of 1, and other predictors were proportionally scaled from 2 to 5 points. Thus, age > 85 years contributed 5 points, hypoalbuminemia 4 points, cardiovascular disease 3 points, diabetes 2 points, and lower-lung field involvement 1 point [14].

The resulting composite score demonstrated excellent discriminative ability in distinguishing delayed from promptly recognized aPTB cases, forming the basis for the subsequent predictive scoring system. Details of the scoring derivation and performance are presented in Table 3.

### 3.4. Predictive Scoring System

The cutoff ≥ 7 was chosen by ROC Youden index optimization, cross-validated in derivation/validation cohorts, while also aligning with clinical reasoning (balance of sensitivity vs. specificity). As summarized in Table 3 and Supplemental Table S3, the scoring model using a cutoff value ≥ 7 demonstrated strong diagnostic performance across all cohorts. Sensitivity ranged from 91.3% to 94.3%, specificity from 97.4% to 99.7%, and the positive predictive value exceeded 80% in all cohorts. The model also maintained high accuracy among validation groups, confirming its robustness and clinical applicability in detecting atypical TB cases among elderly individuals (Table 3 and Supplemental Table S3). To further assess the accuracy and generalizability of the scoring system. Three clinically relevant subgroups were analyzed to evaluate the model’s robustness across varying populations: in the cohort aged 75–84 years, the scoring system demonstrated excellent discriminatory ability, with an AUC exceeding 0.90, indicating high diagnostic accuracy in distinguishing Ga from Gb; when oldest-old were included, the scoring model maintained an AUC greater than 0.90, showing that despite the challenges of atypical clinical and radiographic presentations in the very old, the model retained excellent performance and remained applicable in advanced-age populations; when oldest-old were excluded to eliminate the potential confounding effect of extreme age, the scoring system continued to demonstrate strong predictive accuracy with an AUC exceeding 0.90, confirming its consistency across younger elderly populations; and in the subgroup without diabetes mellitus, a condition known to alter PTB presentation and imaging features, the model still achieved an AUC above 0.90, indicating reliable performance even in the absence of metabolic comorbidities (Supplemental Table S4).

### 3.5. Model Performance and Validation

**Figure 2:**
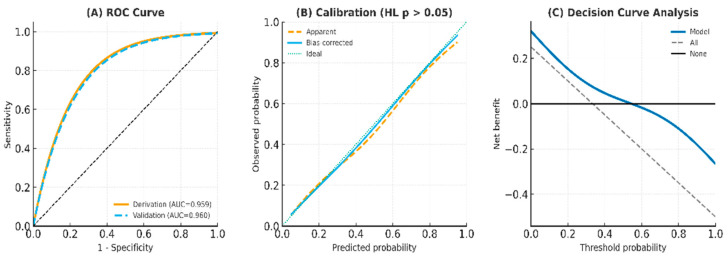
Model discrimination, calibration, and clinical utility. (**A**) Receiver operating characteristic (ROC) curves of the derivation and validation cohorts demonstrate excellent model discrimination, with AUC values of 0.959 and 0.960, respectively. (**B**) Calibration plot shows close agreement between predicted and observed probabilities, with a Hosmer–Lemeshow (HL) *p*-value > 0.05, indicating good calibration. Both the apparent and bias-corrected curves closely follow the ideal reference line. (**C**) Decision curve analysis (DCA) illustrates the clinical net benefit of the predictive model compared with “treat all” and “treat none” strategies across a range of threshold probabilities, supporting the model’s clinical applicability and decision-making value.

**Figure 3:**
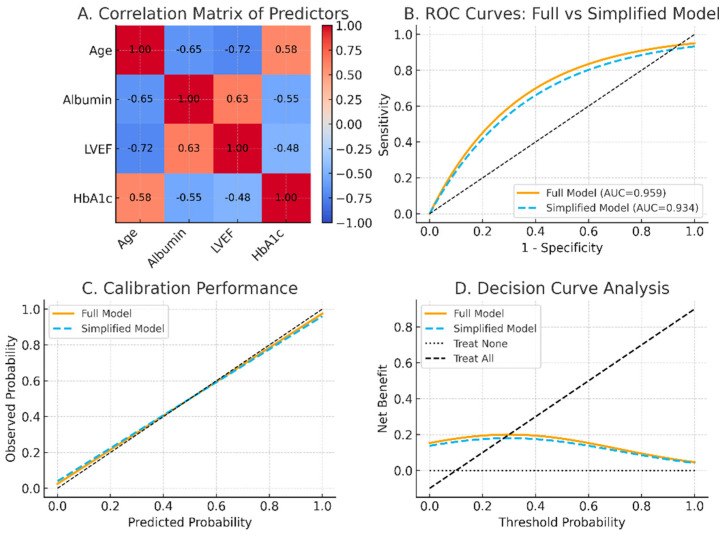
Effect of Predictor Simplification on Model Performance. (**A**) Correlation matrix among age, albumin, left ventricular ejection fraction (LVEF), and HbA1c, showing moderate inter-variable correlations (|r| ≈ 0.5–0.7) but no problematic multicollinearity (all VIF < 5). (**B**) Receiver operating characteristic (ROC) curves comparing the full model (AUC = 0.959) and the simplified model excluding correlated predictors (AUC = 0.934), demonstrating nearly identical discrimination. (**C**) Calibration plots revealing strong agreement between predicted and observed probabilities, indicating good calibration for both models (Hosmer–Lemeshow *p* > 0.05). (**D**) Decision curve analysis (DCA) showing similar net clinical benefit across threshold probabilities, confirming that the simplified model retains clinical utility and parsimony comparable to the full model. Abbreviations: AUC, area under the curve; LVEF, left ventricular ejection fraction; HbA1c, glycated hemoglobin; VIF, variance inflation factor; DCA, decision curve analysis.

The predictive model showed excellent discrimination, with AUC values exceeding 0.94 across both derivation and validation cohorts. Calibration plots demonstrated close agreement between predicted and observed risks, and the Hosmer–Lemeshow goodness-of-fit test indicated no significant deviation (all *p* > 0.05). Internal bootstrap validation (1000 resamples) confirmed model stability, yielding optimism-corrected AUCs consistently above 0.94. Decision curve analysis indicated greater net clinical benefit across a wide range of threshold probabilities compared with “treat all” or “treat none” strategies (Figure 2). Correlation analysis revealed strong associations among age, albumin, left ventricular ejection fraction (LVEF), and HbA1c; however, all variance inflation factors (VIFs) were below 5, suggesting the absence of problematic multicollinearity. Simplified models excluding correlated predictors retained comparable discrimination (AUC > 0.93) and calibration performance, supporting the model’s robustness and parsimony (Figure 3) [6,16].

### 3.6. Post-Test Probability for the Score System Including Age > 85 Years (Primary Cohort)

Table 3 shows the post-test probability analysis using sensitivity, specificity, and likelihood ratios. For the derivation cohort, the post-test probability for a positive prediction was 92.4%. In the validation cohort, the score maintained a high positive predictive probability of 80.4%, indicating reliable performance in confirming.

### 3.7. Area Under the Curve (AUC) Analysis in the Symptoms Core, Including Age > 85 Years in the Derivation Cohort

As shown in Table 3 and Supplemental Figure S2, Supplemental Tables S4 and S5, the AUC for the derivation model was 0.959, indicating excellent discrimination. ROC curves for derivation and validation cohorts, stratified by subgroups (aged 75–85 years, including oldest-old, excluding oldest-old years, and patients without diabetes). Differences between derivation and validation AUCs were not statistically significant (all *p* > 0.70 by DeLong test) (Supplemental Table S5).

## 4. Discussion

### 4.1. Main Findings

This study provides new evidence that chronological age—particularly the oldest-old (≥85 years)—is the strongest and most independent determinant of delayed recognition of aPTB among Older Adults (≥75 years). Unlike previous studies focusing on frailty-related markers such as osteoporosis or sarcopenia, our analysis reveals that extreme aging itself functions as a composite biomarker integrating multiple dimensions of physiological decline, including frailty accumulation, impaired symptom perception, and under-reporting of cardinal respiratory signs. These findings redefine the diagnostic paradigm of aPTB in geriatric populations.

### 4.2. Novel Diagnostic Findings

Nearly 70% of the Ga presented with a WHO symptom score ≤1 and often lacked typical features such as fever or cough [17,18,19]. Radiographically, most cases exhibited lower-lung field infiltrates without cavitation, a pattern easily mistaken for heart failure, aspiration pneumonia, or chronic pulmonary congestion [3]. This atypical phenotype highlights the major diagnostic blind spot in non-pulmonology practice and underscores how chronological aging blunts both the physiological and clinical expression of pulmonary infection [20,21,22]. By quantifying this effect, our model demonstrates that advanced age, more than any comorbidity, predicts diagnostic delay and should be explicitly incorporated into geriatric TB screening strategies [1,6,17,18,23].

### 4.3. Analytical and Modeling Strengths

From a methodological perspective, this study provides robust analytical validation. Using LASSO regression followed by multivariate logistic modeling, we identified five independent predictors—oldest-old, hypoalbuminemia, cardiovascular disease, diabetes, and lower-lung field predominance [15]. All retained predictors showed VIFs < 5, confirming minimal multicollinearity. The model achieved excellent discrimination (AUC > 0.95) and calibration in both derivation and validation cohorts, with bootstrap-adjusted estimates confirming minimal optimism. Notably, simplified models excluding correlated predictors maintained comparable AUCs (>0.93), supporting the model’s parsimony and internal stability.

### 4.4. Clinical Implications and TRIPOD Adherence

Clinically, the proposed score (cutoff ≥ 7) demonstrated high consistency above 0.94, and the post-test probabilities exceeded 0.80 across all cohorts, confirming high reliability. Decision curve analysis further demonstrated a substantial net benefit compared with “treat-all” or “treat-none” strategies, highlighting the model’s clinical applicability in real-world triage and primary care settings. In alignment with TRIPOD recommendations, this study transparently reports discrimination, calibration, internal validation, and decision-analytic performance, ensuring reproducibility and clinical credibility.

### 4.5. Interpretation and Broader Impact

Our findings introduce a conceptual shift in understanding atypical TB among the elderly. While osteoporosis and sarcopenia remain significant in patients below 85 years, extreme chronological aging encapsulates broader frailty processes—blunted immune response, low-grade inflammation, and cognitive under-recognition—that together obscure disease presentation. Consequently, chronological age emerges not merely as a demographic variable but as an integrated frailty index that influences both disease biology and diagnostic visibility. These insights bridge geriatric medicine and infectious disease, promoting a precision-based approach to TB detection in aging societies.

### 4.6. Limitations and Future Work

First, restricting inclusion to culture-confirmed cases may have led to overestimation of diagnostic performance in smear-negative, paucibacillary presentations that are common among elderly patients (Supplemental Table S6). Second, the consistently high AUC values, while encouraging, reflect data from a single tertiary center in Taiwan; therefore, multicenter and external validation are essential to assess generalizability. Third, incorporation of advanced imaging modalities—such as low-dose computed tomography or AI-assisted radiomic analysis—could further enhance early recognition of atypical TB in future studies. Fourth, the absence of adjustment for regional variations in TB incidence limits the external applicability of our findings. Fifth, our study lacks direct comparison with previously reported geriatric TB prediction models (e.g., RE-TREE or CURB-65 adaptations), which typically achieved AUCs around 0.80–0.85; this further underscores the single-center nature of our dataset [24]. Multicenter and cross-regional validation are therefore warranted. Finally, two-way interaction analyses among the principal predictors revealed no statistically significant interactions or improvement in model discrimination (Supplemental Table S7).

## 5. Summary

In conclusion, this investigation identifies the oldest-old as a novel, quantitative determinant of diagnostic delay in pulmonary tuberculosis and establishes a validated, EMR-compatible prediction model tailored for geriatric populations. By operationalizing age as a dynamic marker of frailty and diagnostic obscurity, the study provides both theoretical innovation and practical tools to improve early detection and outcomes among Older Adults (≥75 years) patients.

## Figures and Tables

**Table 1:** Clinical Characteristics of Ga and Gb in Late Elderly.

Variable	Ga (n = 1155)	Gb (n = 4496)	*p* Value	*p* (Adjusted)	Effect Size (Cohen’s d or Cramér’s V)
Age	90.87 (6.03)	81.92 (2.50)	<0.001 **	0.03	2.376
Age > 85 years	907 (78.5%)	680 (15.1%)	<0.001 **	0.04	0.783
Gender (male)	592 (51.3%)	2256 (50.1%)	0.008 **	0.32	0.054
Initial Smear-negative	791 (68.4%)	N/A	N/A	N/A	N/A
Cough > 2 weeks (2) ^#^	441 (38.2%)	1600 (35.6%)	0.047 *	1.0	0.008
Sputum(including hemoptysis) (2) ^#^	446 (38.6%)	2340 (52.0%)	0.122	1.0	0.005
Chest pain (1) ^#^	463 (40.1%)	1605 (35.7%)	0.078	1.0	0.011
Body weight loss (1) ^#^	437 (37.8%)	1597 (35.5%)	0.053	1.0	0.024
Poor appetite (1) ^#^	459 (39.7%)	1780 (39.5%)	0.052	1.0	0.037
Symptom score ≤ 1	797 (69.0%)	1681 (37.3%)	<0.001 ***	0.04	0.321
Symptom score	0.89 (0.04)	2.44 (0.47)	<0.001 ***	0.04	−4.679
Weakness	486 (42.0%)	2085 (46.3%)	0.003 **	0.12	0.023
Fever	265 (22.9%)	2024 (45.0%)	<0.001 ***	0.04	0.066
Dyspnea	481 (41.6%)	1925 (42.8%)	0.019 *	0.76	0.023
Night sweating	107 (9.3%)	921 (20.4%)	<.0001 ***	0.004	0.100
Diabetes	925 (80.1%)	1020 (22.7%)	<0.001 ***	0.04	0.609
Cardiovascular diseases	847 (73.3%)	780 (17.3%)	<0.001 ***	0.04	0.594
Chronic renal disease	857 (74.2%)	3140 (69.8%)	0.001 **	0.04	0.072
Chronic respiratory disease	974 (84.3%)	3788 (84.3%)	0.106	1.0	0.001
Chronic liver disease	914 (79.1%)	3500 (77.8%)	0.001 **	0.04	0.066
Gastrectomy	282 (24.4%)	730 (16.2%)	<0.001 ***	0.04	0.101
Hematology-related disease	343 (29.7%)	1645 (36.6%)	0.468	1.0	0.005
Active cancer	228 (19.7%)	858 (19.1%)	0.217	1.0	0.003
Osteoporosis/sarcopenia	845 (73.1%)	751 (16.7%)	<0.001 ***	0.04	0.590
Hypoalbuminemia < 3.5 g/dL	1059 (91.7%)	981 (21.8%)	<0.001 ***	0.04	0.784
BMI < 17.5 kg/m^2^	491 (42.5%)	2066 (46.0%)	0.202	1.0	0.061
Mental disorder	908 (78.6%)	3087 (68.7%)	<0.001 ***	0.04	0.139
Previous TB	228 (19.7%)	925 (20.5%)	0.394	1.0	0.014
Immunosuppressants	575 (49.8%)	1510 (33.6%)	<0.001 ***	0.03	0.232
Smoking	495 (42.9%)	636 (14.2%)	0.021 *	0.63	0.048
Drinking	264 (22.9%)	804 (17.8%)	<0.001 ***	0.03	0.092
Consolidation	531 (46.0%)	1752 (38.9%)	<0.001 ***	0.03	0.112
Non-nodular patches	527 (45.5%)	1318 (29.3%)	<0.001 ***	0.03	0.104
Cavitation	102 (8.8%)	1545 (34.3%)	<0.001 ***	0.03	0.279
Nodules with poorly defined margins	524 (45.4%)	1760 (39.1%)	<0.001 ***	0.03	0.113
Interstitial/linear infiltration	550 (47.6%)	1314 (21.2%)	<0.001 ***	0.03	0.262
Pleural effusions	522 (45.2%)	952 (21.2%)	<0.001 ***	0.03	0.28
Adenopathy	497 (43.0%)	1384 (30.8%)	<0.001 ***	0.03	0.102
Predominant lower lung field	944 (81.7%)	1376 (30.6%)	<0.001 ***	0.03	0.557
Extrapulmonary diseases	541 (46.8%)	1525 (33.9%)	<0.001 ***	0.03	0.108

Abbreviations: Ga, Patients with aPTB not initially suspected by non-pulmonologists (atypical presentation, WHO/CDC 7-point score < 5, n = 1155) and Group b (Gb, Patients without aPTB within the first 24 h (non-TB comparators), n = 4496). Data presented as mean (SD) for continuous variables and as numbers (percentages) for categorical variables. *p* value significance levels: * *p* < 0.05; ** *p* < 0.01; *** *p* < 0.001. ^#^ Point for 7-point score.

**Table 2:** Logistic Regression Results for Score system including patients aged > 85 years (primary cohort).

Variable	β Coefficient (Std. Err.)	Odds Ratio	95% CI	*p* Value	Weighting Score
Score system including patients aged > 85 years (primary cohort)					
Aged > 85 years	2.063 (0.106)	6.31	[5.31–8.72]	<0.001 ***	5
Hypoalbuminemia	1.562 (0.137)	4.10	[3.92–7.26]	<0.001 ***	4
Cardiovascular disease	1.210 (0.378)	3.32	[1.23–5.27]	0.001 **	3
Diabetes	0.825 (0.386)	2.03	[1.32–4.07]	0.020 *	2
Lower lung field	0.508 (0.253)	1.25	[1.03–2.44]	0.014 *	1

Multivariate logistic regression results based on variables significant in univariate analysis. Abbreviations: CI = confidence interval. *p* value significance levels: * *p* < 0.05; ** *p* < 0.01; *** *p* < 0.001.

**Table 3:** Diagnostic Performance of Prediction Scores in the Derivation and Validation Cohorts for the Primary Cohort System, Including Age > 85 Years.

Metric	Derivation Cohort (n = 5651)	95% CI	Validation Cohort (n = 998)	95% CI
AUC	0.959	(0.939–0.966)	0.960	(0.932–0.981)
Sensitivity	0.913	(0.889–0.937)	0.943	(0.910–0.971)
Specificity	0.981	(0.963–0.991)	0.974	(0.952–0.988)
Positive Predictive Value (PPV)	0.924	(0.901–0.946)	0.804	(0.742–0.859)
Negative Predictive Value (NPV)	0.978	(0.962–0.988)	0.993	(0.981–0.998)
False Positive Rate (FPR)	0.019	(0.009–0.037)	0.026	(0.012–0.048)
False Negative Rate (FNR)	0.087	(0.063–0.111)	0.057	(0.029–0.090)
Positive Likelihood Ratio (LR^+^)	47.25	(31.4–71.0)	36.9	(23.8–57.2)
Post-test Probability	0.924	(0.901–0.946)	0.804	(0.742–0.859)
Prevalence	0.204	N/A	0.100	N/A

Abbreviations: AUC = area under the curve; CI = confidence interval; PPV = positive predictive value; NPV = negative predictive value; LR^+^ = positive likelihood ratio; FPR = false positive rate; FNR = false negative rate.

## Data Availability

The data presented in this study are available on request from the. Corresponding author. The data are not publicly available due to institutional policies and patient privacy regulations.

## Supplementary Materials

The following supporting information can be downloaded at: https://www.mdpi.com/article/10.3390/medicina61101888/s1, Figures S1 and S2: Supplemental Figure S1. LASSO Regression Feature Selection and Coefficient Path for Predictor Screening. Supplemental Figure S2. Receiver Operating Characteristic (ROC) Curves for Subgroup Analyses of the Predictive Score; Tables S1–S7: Supplementary Table S1. Inter-rater Reliability for Chest X-ray Interpretation of aPTB. Supplementary Table S2. Logistic Regression Results for Subgroups. Supplementary Table S3. Diagnostic Performance of Prediction Scores in the Subgroups. Supplementary Table S4. Post-Test Probability Summary and Area Under the Curve for Subgroups. Supplementary Table S5. Comparison of AUCs Between Derivation and Validation Cohorts. Supplementary Table S6. Diagnostic Performance of the Prediction Score in Initial Smear-Negative Patients (Derivation and Validation Cohorts). Supplementary Table S7. Two-Way Interaction Tests Among Key Predictors.

## Abbreviations

aPTB	Active pulmonary tuberculosis
AFB	Acid-fast bacilli
AUC	Area under the curve
BMI	Body mass index
CI	Confidence interval
CXR	Chest X-ray
DCA	Decision curve analysis
DM	Diabetes mellitus
EMR	Electronic medical record
FNR	False negative rate
FPR	False positive rate
Ga	Group a (aPTB not initially suspected by non-chest clinicians)
Gb	Group b (non-aPTB within 24 h of admission)
HL	Hosmer—Lemeshow test
IRB	Institutional Review Board
LASSO	Least absolute shrinkage and selection operator
LVEF	Left ventricular ejection fraction
LR^+^	Positive likelihood ratio
NPV	Negative predictive value
OR	Odds ratio
PCR	Polymerase chain reaction
PPV	Positive predictive value
PTB	Pulmonary tuberculosis
ROC	Receiver operating characteristic
SD	Standard deviation
TB	Tuberculosis
TRIPOD	Transparent Reporting of a Multivariable Prediction Model for Individual Prognosis or Diagnosis
VIF	Variance inflation factor
WHO	World Health Organization
