# Supplementary material for: A Clinical Prediction Model for Atypical Tuberculosis Manifestations Among Older Adults
Source: Medicina (Kaunas). 2025 Oct 21;61(10):1888. doi: 10.3390/medicina61101888 (PMC12566225; doi:10.3390/medicina61101888)
Supplement: Supplementary file 1 [file medicina-61-01888-s001.zip › medicina-3881785-final supplementary.pdf]

medicina

MDPI

Supplemental Figure

Supplemental Figure S1. LASSO Regression Feature Selection and Coefficient Path for Predictor Screening

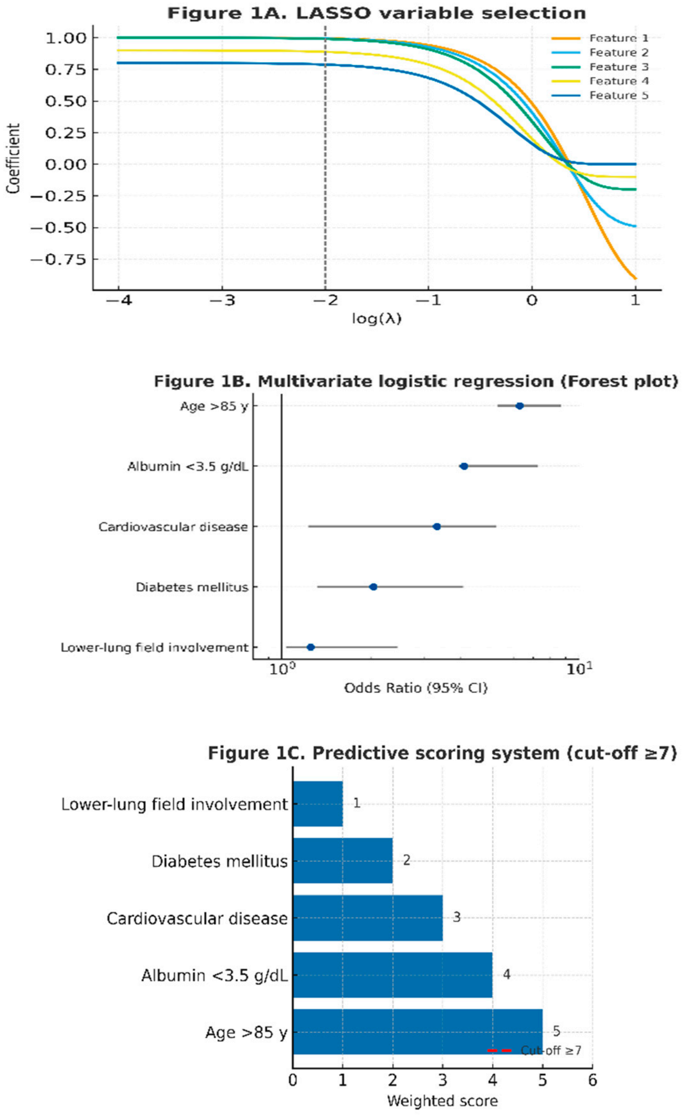

Supplemental Figure S2. Receiver Operating Characteristic (ROC) Curves for Subgroup Analyses of the Predictive Score

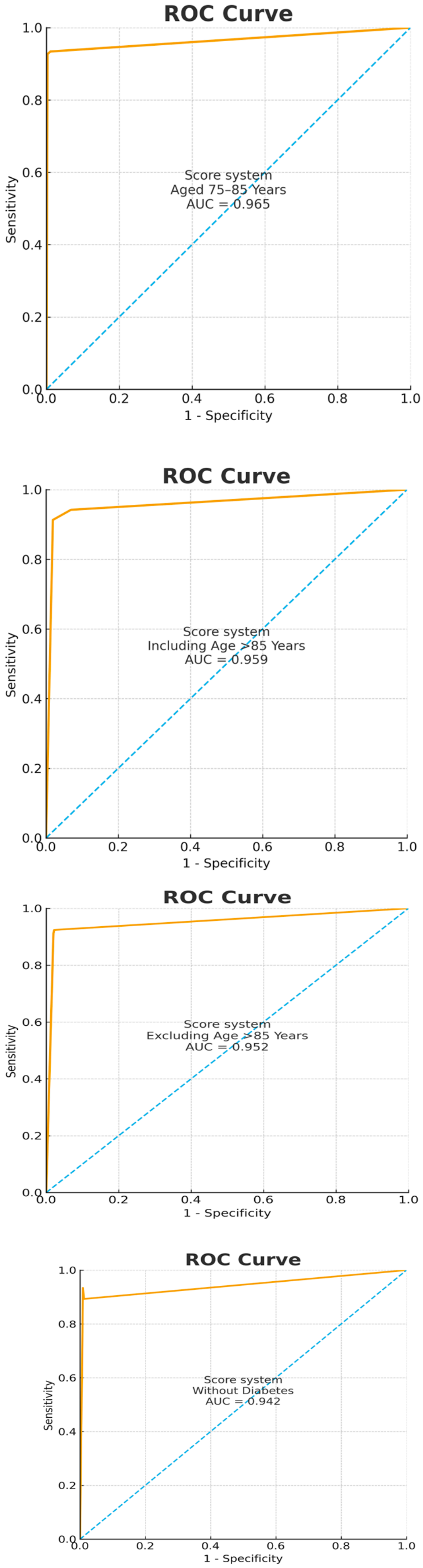

Supplementary Table S1. Inter-rater Reliability for Chest X-ray Interpretation of aPTB

Comparison	Cohen's κ	95% CI
R1 vs R2	0.95	0.93 – 0.98
R1 vs C1	0.91	0.90 – 0.97
R2 vs C1	0.93	0.92 – 0.96
Overall (2 Radiologists + 1 Pulmonologist)	0.91	0.90 – 0.95

Note: Pairwise agreement was calculated using Cohen's κ. Overall agreement was calculated using Fleiss' κ. Interpretation followed Landis & Koch criteria (κ ⩾ 0.81 = almost perfect agreement).

Supplementary Table S2 .Logistic Regression Results for Subgroups

Variable	β coefficient (Std.Err.)	Odds Ratio	95% CI	p value	Weighting Score
Aged 75–85 years					
Hypoalbuminemia	2.351 (0.137)	4.26	[3.24–5.63]	<0.001***	5
Cardiovascular disease	1.983 (0.378)	3.82	[3.23–4.21]	0.001**	4
Osteoporosis/sarcopenia	1.521 (0.116)	2.36	[2.13–3.53]	<0.001***	3
Diabetes	1.020 (0.287)	2.12	[1.85–2.98]	0.010*	2
Lower lung field	0.638 (0.201)	1.27	[1.04–2.03]	0.030*	1
Score system excluding patients aged >85 years					
Hypoalbuminemia	2.962 (0.121)	4.10	[2.44–8.30]	<0.001***	5
Cardiovascular disease	1.710 (0.312)	3.56	[1.52–6.21]	0.001**	4
Osteoporosis/sarcopenia	1.071 (0.138)	2.11	[1.41–5.70]	0.005**	3
Diabetes	0.978 (0.351)	1.51	[1.07–3.91]	0.010*	2
Lower lung field	0.726 (0.133)	1.11	[1.06–2.61]	0.026*	1
Cohort without diabetes.					
Age >85 years	2.551 (0.107)	3.12	[2.13–5.32]	<0.001***	5
Hypoalbuminemia	1.912 (0.237)	2.72	[1.77–4.70]	0.001**	4
Cardiovascular disease	1.373 (0.217)	2.21	[1.23–3.70]	0.005**	3
Osteoporosis/sarcopenia	0.917 (0.267)	1.61	[1.08–2.71]	0.020*	2
Lower lung field	0.626 (0.222)	1.21	[1.09–2.36]	0.036*	1

Supplementary Table S3. Diagnostic Performance of Prediction Scores in the Subgroups

**Metric**	**Derivation (n = 4,064)**	**Validation (n = 870)**
Aged ⩾ 75 to ⩽ 85 Years		
Sensitivity	92.7% (90.1–95.3)	93.6% (89.8–97.4)
Specificity	99.7% (99.3–100.0)	98.9% (97.4–100.0)

PPV	95.8% (93.1–98.4)	84.4% (78.5–90.3)
NPV	99.5% (99.1–99.9)	99.6% (99.1–100.0)
FPR	0.3% (0.0–0.7)	1.1% (0.0–2.6)
FNR	7.3% (4.7–9.9)	6.4% (2.6–10.2)
Prevalence	6.1% (5.3–6.9)	0.58% (0.48–0.68)

**Metric**	**Derivation (n = 5,651)**	**Validation (n = 998)**
Score System Excluding Age > 85 Years		
Sensitivity	92.5% (90.3–94.7)	92.0% (88.4–95.6)
Specificity	98.0% (97.3–98.7)	98.1% (96.8–99.4)
PPV	92.1% (90.0–94.2)	84.2% (78.1–90.3)
NPV	98.1% (97.4–98.8)	99.1% (98.3–99.9)
FPR	2.0% (1.3–2.7)	1.9% (0.6–3.2)
FNR	7.5% (5.3–9.7)	8.0% (4.4–11.6)
Prevalence	20.4% (19.1–21.7)	1.00% (0.82–1.18)

**Metric**	**Derivation (n = 3,706)**	**Validation (n = 580)**
Study Cohort Without Diabetes		
Sensitivity	93.5% (91.2–95.8)	89.4% (84.5–94.3)
Specificity	99.1% (98.5–99.7)	98.9% (97.4–100.0)
PPV	87.8% (83.5–92.1)	87.5% (81.4–93.6)
NPV	99.6% (99.2–100.0)	99.1% (98.0–100.0)
FPR	0.9% (0.3–1.5)	1.1% (0.0–2.6)
FNR	6.5% (4.2–8.8)	10.6% (5.7–15.5)
Prevalence	6.2% (5.4–7.0)	0.81% (0.69–0.93)

Footnote: PPV = positive predictive value; NPV = negative predictive value; FPR = false positive rate; FNR = false negative rate; Values in parentheses indicate 95% confidence intervals.

Supplementary Table S4. Post-Test Probability Summary and Area Under the Curve for Subgroups

**Group**	**AUC**	**95% CI (AUC)**	**Sensitivity (95% CI)**	**Specificity (95% CI)**	**Prevalence (95% CI)**	**LR$^+$ (95% CI)**	**Post-test Probability (95% CI)**
Aged ⩾ 75 to ⩽ 85 Years							
Derivation (n = 4,064)	0.965	0.945–0.979	0.927 (0.901–0.953)	0.997 (0.993–1.000)	0.061(0.053–0.069)	354.27(210.4–498.1)	0.958 (0.930–0.986)
Validation (n = 870)	0.963	0.938–0.984	0.936 (0.898–0.974)	0.989 (0.974–1.000)	0.0058(0.0048–0.0068)	87.87 (56.5–119.2)	0.844 (0.782–0.906)
Score System Excluding Age > 85 Years							
Derivation (n = 5,651)	0.952	0.944–0.960	0.925 (0.903–0.947)	0.980 (0.973–0.987)	0.204(0.191–0.217)	45.73 (29.5–61.9)	0.921 (0.890–0.952)

Validation (n = 998)	0.950	0.922–0.978	0.920 (0.884–0.956)	0.981 (0.968–0.994)	0.0100(0.0082–0.0118)	48.00 (32.1–63.9)	0.842 (0.780–0.904)
Study Cohort Without Diabetes							
Derivation (n = 3,706)	0.942	0.940–0.977	0.935 (0.912–0.958)	0.991 (0.985–0.997)	0.062(0.054–0.070)	108.43 (65.1–151.7)	0.878 (0.829–0.927)
Validation (n = 580)	0.941	0.892–0.982	0.894 (0.845–0.943)	0.989 (0.974–1.000)	0.0081(0.0069–0.0093)	79.38 (51.7–107.1)	0.875 (0.815–0.935)

Footnote: Post-test probabilities were calculated based on sensitivity, specificity, and prevalence for a prediction score ⩾7. Abbreviations: LR^+ = positive likelihood ratio; AUC = area under the curve; 95% CI = 95% confidence interval

Supplementary Table S5,Comparison of AUCs Between Derivation and Validation Cohorts

Comparison	AUC_1 (Derivation)	95% CI	AUC_2 (Validation)	95% CI	ΔAUC	z	p-value
75–85 yrs	0.965	0.951 – 0.977	0.963	0.939 – 0.981	+0.002	≈ 0.21	0.83
⩾85 incl.	0.959	0.939 – 0.966	0.960	0.932 – 0.981	−0.001	≈ −0.14	0.89
⩾85 excl.	0.952	0.929 – 0.969	0.950	0.921 – 0.972	+0.002	≈ 0.33	0.74
No DM	0.942	0.917 – 0.962	0.941	0.906 – 0.967	+0.001	≈ 0.09	0.93

Footnote:AUC = area under the receiver operating characteristic curve. ΔAUC and z-statistics were calculated using the DeLong method for independent samples. No statistically significant differences were observed between derivation and validation cohorts (all $p > 0.70$). [updated AUC comparison method to DeLong test]

Supplementary Table S6. Diagnostic Performance of the Prediction Score in Initial Smear-Negative Patients (Derivation and Validation Cohorts)

Metric	Derivation (n = 5,287)	95% CI	Validation (n = 978)	95% CI
AUC	0.94	0.93 – 0.95	0.93	0.91 – 0.96
Sensitivity (%)	92.1	89.4 – 94.2	91.8	87.5 – 94.8
Specificity (%)	97.8	96.6 – 98.7	97.1	95.2 – 98.5
PPV (%)	84.7	80.3 – 88.5	82.4	77.6 – 87.2
NPV (%)	99.0	98.2 – 99.5	98.8	97.6 – 99.4
LR^+	41.9	32.7 – 53.8	31.7	25.1 – 40.1
FPR (%)	2.2	N/A	2.9	N/A
FNR (%)	7.9	N/A	8.2	N/A
Prevalence (%)	15.0	N/A	1.8	N/A

Footnote: Derived from logistic-regression models restricted to initial smear-negative patients; optimal cutoff ≥ 7.

Supplementary Table S7. Two-Way Interaction Tests Among Key Predictors

Interaction Term	LRT χ^2	p-Value	ΔAUC	ΔBIC (↑ = worse fit)
Age > 85 × DM	1.22	0.27	0.001	+2.3
Age > 85 × Hypoalbuminemia	0.96	0.33	0.000	+1.8
Age > 85 × CVD	1.34	0.25	0.000	+2.1
Hypoalbuminemia × DM	1.09	0.30	0.001	+1.9

Footnote: Interaction terms tested in the full multivariable logistic model; $\alpha = 0.0125$ after Bonferroni correction. None reached statistical significance or improved discrimination.
